# Identification of New Factors Modulating Adhesion Abilities of the Pioneer Commensal Bacterium *Streptococcus salivarius*

**DOI:** 10.3389/fmicb.2018.00273

**Published:** 2018-02-20

**Authors:** Benoit Couvigny, Saulius Kulakauskas, Nicolas Pons, Benoit Quinquis, Anne-Laure Abraham, Thierry Meylheuc, Christine Delorme, Pierre Renault, Romain Briandet, Nicolas Lapaque, Eric Guédon

**Affiliations:** ^1^MICALIS Institute, INRA, AgroParisTech, Université Paris-Saclay, Jouy-en-Josas, France; ^2^MetaGenoPoliS, INRA, Université Paris-Saclay, Jouy-en-Josas, France; ^3^MaIAGE, INRA, Jouy-en-Josas, France; ^4^INRA, Plateforme MIMA2, Jouy-en-Josas, France; ^5^STLO, UMR 1253, INRA, Agrocampus Ouest, Rennes, France

**Keywords:** adhesion gene, biofilm formation, aggregation, sedimentation, host interactions, commensal bacteria, *Streptococcus salivarius*

## Abstract

Biofilm formation is crucial for bacterial community development and host colonization by *Streptococcus salivarius*, a pioneer colonizer and commensal bacterium of the human gastrointestinal tract. This ability to form biofilms depends on bacterial adhesion to host surfaces, and on the intercellular aggregation contributing to biofilm cohesiveness. Many *S. salivarius* isolates auto-aggregate, an adhesion process mediated by cell surface proteins. To gain an insight into the genetic factors of *S. salivarius* that dictate host adhesion and biofilm formation, we developed a screening method, based on the differential sedimentation of bacteria in semi-liquid conditions according to their auto-aggregation capacity, which allowed us to identify twelve mutations affecting this auto-aggregation phenotype. Mutations targeted genes encoding (i) extracellular components, including the CshA surface-exposed protein, the extracellular BglB glucan-binding protein, the GtfE, GtfG and GtfH glycosyltransferases and enzymes responsible for synthesis of cell wall polysaccharides (CwpB, CwpK), (ii) proteins responsible for the extracellular localization of proteins, such as structural components of the accessory SecA2Y2 system (Asp1, Asp2, SecA2) and the SrtA sortase, and (iii) the LiaR transcriptional response regulator. These mutations also influenced biofilm architecture, revealing that similar cell-to-cell interactions govern assembly of auto-aggregates and biofilm formation. We found that BglB, CshA, GtfH and LiaR were specifically associated with bacterial auto-aggregation, whereas Asp1, Asp2, CwpB, CwpK, GtfE, GtfG, SecA2 and SrtA also contributed to adhesion to host cells and host-derived components, or to interactions with the human pathogen *Fusobacterium nucleatum*. Our study demonstrates that our screening method could also be used to identify genes implicated in the bacterial interactions of pathogens or probiotics, for which aggregation is either a virulence trait or an advantageous feature, respectively.

## Introduction

*Streptococcus salivarius* is one of the early colonizers of oral mucosa surfaces in neonates and is a commensal inhabitant of the oral cavity and digestive tract of healthy adults. *S. salivarius* is thought to exert a range of biological activities related to host health, in particular through its impact on the stability of microbiota composition, and its interaction with the host (Delorme et al., 2015). Despite the role of *S. salivarius* in both oral and digestive tract ecology, the factors that allow this bacterium to become established and then maintained in the host environment have not yet been the subject of extensive molecular and genetic analyses.

Bacterial adhesion is an initial and critical step in the oral colonization process. Adhesion processes may include attachment of the bacterial cell to host cells, to components of the extracellular matrix (ECM), to the salivary pellicle on teeth and to soluble factors, as well as to bacterial cells of the same strain (auto-aggregation) or genetically distinct species (co-aggregation). The ability of *S. salivarius* to colonize and maintain at multiple niches throughout the lifespan of its host suggests that this bacterium has evolved various adhesive strategies (Delorme et al., 2015). Numerous studies have described the remarkable capability of *S. salivarius* to bind to a wide range of biological surfaces, including buccal, hypopharyngeal, bronchial and cervicovaginal epithelial cell lines (Handley et al., 1987; Cosseau et al., 2008; Guglielmetti et al., 2010; Burton et al., 2013), various proteins in human saliva and of the ECM (Kilian and Nyvad, 1990; Schenkels et al., 1993; Couvigny et al., 2017) and to MUC2, a glycoprotein found in saliva and on mucosal surfaces of the ileum and colon (Kilian and Nyvad, 1990; Schenkels et al., 1993; McGuckin et al., 2011). *S. salivarius* is also able to form auto-aggregates (Couvigny et al., 2017) and to co-aggregate with numerous oral microorganisms such as the early colonizers *Veillonella* (Weerkamp and McBride, 1980, 1981; Handley et al., 1987) and *Prevotella* species (Levesque et al., 2003), the intermediate colonizers *Fusobacterium nucleatum* (Weerkamp and McBride, 1980; Levesque et al., 2003) and *Candida albicans* (Nikawa et al., 2001; Levesque et al., 2003), and the late colonizers *Tannerella forsythia* (Shimotahira et al., 2013) and *Porphyromonas gingivalis* (Levesque et al., 2003).

Our knowledge of the factors mediating adhesion of *S. salivarius* to biological surfaces remains limited. It has been shown that cell wall-associated fimbriae and fibrils are involved in this adhesion process. Indeed, fimbriae are responsible for adhesion to cervicovaginal epithelial cells and co-aggregation with *Prevotella intermedia* (Levesque et al., 2003; Burton et al., 2013), whereas fibrils mediate co-aggregation with *Veillonella* species and adhesion to various host surfaces (Weerkamp and McBride, 1981; Weerkamp and Jacobs, 1982). However, the genes encoding the constituent proteins of these structures remain to be identified. Recently, we showed that SrpB and SrpC, two adhesins belonging to the SRR (Serine-rich repeat) glycoprotein family, mediate auto-aggregation and adhesion to a wide range of epithelial cells and ECM proteins (Couvigny et al., 2017). SrpB/C proteins are glycosylated by the GtfE/F glycosyltransferases and are secreted through a dedicated transport system (i.e., the accessory SecA2/Y2 system) to the cell surface where they form fibril-like structures. Genes involved in SRR transport are located in the conserved *secA2*/*Y2* genomic cluster, encoding the SecA2 motor protein, the SecY2 membrane translocation complex, the Asp1/2/3/4/5 chaperones and the GtfA/B/C/D glycosyltransferases. Components of the SecA2/Y2 system and GtfE/F are required for adhesion of *S. salivarius* to ECM and to epithelial cells and for auto-aggregation through their activities on SrpB/C (Couvigny et al., 2017).

The objective of this study was to identify novel factors that influence the ability of *S. salivarius* to form biofilms and host–cell interactions. Measurements of auto-aggregation are widely used as a method for evaluating the adhesion capacity of bacteria. We developed a strategy, based on immobilization of cell clusters in semi-liquid agar medium, to select mutants with increased and decreased auto-aggregation phenotypes. We then identified the genes involved and evaluated the ability of the auto-aggregation mutants to bind various host surfaces, to co-aggregate with *F. nucleatum* and to form biofilms.

## Materials and Methods

### Bacterial Strains, Growth Conditions, and DNA Manipulation

Bacterial strains used in this study are listed in **Table 1**. *S. salivarius* strains were grown as described previously (Couvigny et al., 2015b). *F. nucleatum* DSM 20482 was grown in brain heart infusion broth supplemented with 0.25% L-glutamic acid. Erythromycin (5 μg/ml) or kanamycin (1000 μg/ml) were added to the medium as required. PCRs were performed using Phusion^®^ high-fidelity DNA polymerase (New England Biolabs, Ipswich, MA, United States). The primers were purchased from Eurofins MWG Operon (Germany) and are listed in Supplementary Table S1. When necessary, PCR products and DNA restriction fragments were purified using QIAquick kits (Qiagen). Plasmids were purified using the QIAprep Miniprep kit (Qiagen).

**Table 1:** Bacterial strains.

Strain	Relevant characteristics^(a)^	Reference or source
JIM8777	*Streptococcus salivarius* wild-type strain, Ag^+^ phenotype	Guedon et al., 2011
JIM9442	*cwpB_spont_*, spontaneous JIM8777 mutant, frameshift mutation in SALIVA_1034, Ag^++^ phenotype	This work
JIM9443	*cwpK_spont_*, spontaneous JIM8777 mutant, nonsense mutation in SALIVA_1043 (R342opal), Ag^++^ phenotype	This work
JIM9445	*asp1*_*spont*1_, spontaneous JIM8777 mutant, nonsense mutation in SALIVA_1467 (E98ochre), Ag^-^ phenotype	This work
JIM9446	*asp1*_*spont*2_, spontaneous JIM8777 mutant, nonsense mutation in SALIVA_1467 (E213ochre), Ag^-^ phenotype	This work
JIM9447	*asp2_spont_*, spontaneous JIM8777 mutant, frameshift mutation in SALIVA_1466, Ag^-^ phenotype	This work
JIM9448	*liaR_spont_*, spontaneous JIM8777 mutant, missense mutation in SALIVA_1496 (M15I), Ag^-^ phenotype	This work
JIM9310	Δ*asp1*, JIM8777 ΔsalivA_1467::*erm*, Ag^-^ phenotype	Couvigny et al., 2017
JIM9324	Δ*asp1*, JIM8777 ΔsalivA_1467::*kan*, Ag^-^ phenotype	This work
JIM9307	Δ*asp2*, JIM8777 ΔsalivA_*1466*::*erm*, Ag^-^ phenotype	Couvigny et al., 2017
JIM9478	Δ*bglB*, JIM8777 ΔsalivA_0826::*erm*, Ag^+^ phenotype	This work
JIM9465	Δ*cwpK*, JIM8777 ΔsalivA_1043::*erm*, Ag^++^ phenotype	This work
JIM9469	Δ*cwpK*Δ*asp1*, JIM9324 ΔsalivA_1043::*erm*, Ag^-^ phenotype	This work
JIM9486	Δ*gtfE*, JIM8777 ΔsalivA_0390::*erm*, Ag^-^ phenotype	Couvigny et al., 2017
JIM9482	Δ*cshA*, JIM8777 ΔsalivA_0893::erm, Ag^-^ phenotype	This work
JIM9490	Δ*gtfG*, JIM8777 ΔsalivA_1698::*erm*, Ag^-^ phenotype	This work
JIM9492	Δ*gtfH*, JIM8777 ΔsalivA_1700::*erm*, Ag^-^ phenotype	This work
JIM9471	Δ*liaR*, JIM8777 ΔsalivA_1496::*erm*, Ag^-^ phenotype	This work
JIM9303	Δ*secA2*, JIM8777 ΔsalivA_1464::*erm*, Ag^-^ phenotype	Couvigny et al., 2017
JIM9496	Δ*srtA*, JIM8777 ΔsalivA_1273::*erm*, Ag^-^ phenotype	This work
CCHSS1	*Streptococcus salivarius*, Human blood	Delorme et al., 2007
CCHSS2	*Streptococcus salivarius*, Human blood	Delorme et al., 2007
CCHSS3	*Streptococcus salivarius*, Human blood	Delorme et al., 2011
CCHSS4	*Streptococcus salivarius*, Human blood	Delorme et al., 2007
CCHSS7	*Streptococcus salivarius*, Human blood	Delorme et al., 2007
CIP104994	*Streptococcus salivarius*, Human blood	Delorme et al., 2007
JIM8223	*Streptococcus salivarius*, Oral cavity	Delorme et al., 2007
JIM8421	*Streptococcus salivarius*, Breast milk	Delorme et al., 2007
JIM8771	*Streptococcus salivarius*, Oral cavity	Delorme et al., 2007
JIM8772	*Streptococcus salivarius*, Oral cavity	Delorme et al., 2007
JIM8773	*Streptococcus salivarius*, Oral cavity	Delorme et al., 2007
JIM9074	*Streptococcus salivarius*, Human blood	Couvigny et al., 2015a
JIM9075	*Streptococcus salivarius*, Human blood	This work
JIM9076	*Streptococcus salivarius*, Sputum	Couvigny et al., 2015a
JIM9080	*Streptococcus salivarius*, Peritoneal cavity	Couvigny et al., 2015a
JIM9082	*Streptococcus salivarius*, Peritoneal cavity	Couvigny et al., 2015a
JIM9086	*Streptococcus salivarius*, Peritoneal cavity	Couvigny et al., 2015a
JIM9087	*Streptococcus salivarius*, Human blood	Couvigny et al., 2015a
JIM9089	*Streptococcus salivarius*, Human blood	Couvigny et al., 2015a
JIM9090	*Streptococcus salivarius*, Human blood	This work
JIM9091	*Streptococcus salivarius*, Peritoneal cavity	This work
JIM9092	*Streptococcus salivarius*, Human blood	This work
JIM9095	*Streptococcus salivarius*, Human trachea	This work
JIM9096	*Streptococcus salivarius*, Lower left lung	This work
JIM9101	*Streptococcus salivarius*, Human blood	This work
JIM9102	*Streptococcus salivarius*, Peritoneal cavity	Couvigny et al., 2015a
JIM9104	*Streptococcus salivarius*, Human blood	This work
DSM 20482	*Fusobacterium nucleatum* subsp. *polymorphum*, type strain	DSMZ


*^*(a)*^Ag, auto-aggregation.*

### Isolation of Mutants with Altered Sedimentation Properties

Mutants with altered auto-aggregation properties were isolated using Todd Hewitt broth with 0.5% yeast extract with a low (0.03%) concentration of agar (Mercier et al., 2000, 2002; Llull et al., 2005). In these conditions, the extent of free displacement of bacteria in the environment depends on the viscosity of the semi-liquid (SL) medium.

### Genome-Wide Mutagenesis and Characterization of Transposon Targets

Mutagenesis with pGhost9::IS*S1* was performed essentially as described previously (Guedon et al., 2001). Briefly, cells containing pGhost9::IS*S1* were grown overnight at 30°C in M17 medium in the presence of erythromycin. Stationary-phase cultures were diluted 1:1000 in fresh M17 broth without erythromycin, incubated for 150 min at 30°C and then at 38°C for 150 min. Samples were then diluted and plated at 38°C. Clones that grew on M17 medium containing erythromycin were selected as transposon mutants. Approximately 10,000 mutants were pooled and the mixture was deposited on the surface of 20 flasks containing SL medium and erythromycin. After 140 h of growth, 40 samples at the ends of the roots were removed and streaked out on agar plates to isolate single colonies of auto-aggregation mutants. The pGhost9::IS*S1* insertion site was identified by cloning and sequencing of the chromosomal junctions as described previously (Couvigny et al., 2015b).

### Whole-Genome Sequencing of Spontaneous Auto-aggregation Mutant Strains

*Streptococcus salivarius* spontaneous mutants JIM9443 and JIM9447 were sequenced using the Illumina HiSeq 2000 system at I2BC^1^, with around 7 million paired-end 100 base-long reads. Strains JIM9442, JIM9445, JIM9446, and JIM9448 were sequenced using the SOliD technology 4 platform at MetaGenoPoliS^2^, with around 5 million single 50 base-long reads. SOLiD reads containing adapter and/or barcode fragments with a mean quality value <20 and low-quality reads (with 3 or more “N”) were discarded. Clean reads were mapped to the reference JIM8777 strain using Bowtie (Langmead et al., 2009) and variations were detected using the Tablet software (Milne et al., 2013). The Illumina and SOLiD sample sequences are available in the SRA database (project ID PRJEB23560).

### Construction of Deletion Strains by Natural Transformation

Mutant derivatives of strain JIM8777 were constructed by exchanging the coding sequence of a target gene (sequence between the start and stop codon) for an erythromycin or kanamycin resistance cassette devoid of a transcriptional regulatory region (e.g., promoter or terminator), as described previously (Couvigny et al., 2015b). After peptide-induced transformation, integration of the antibiotic cassette at the appropriate location was verified by PCR (Supplementary Table S1).

### Adhesion Assay

Adhesion of *S. salivarius* strains to HT-29 human epithelial cell lines (colon adenocarcinoma; ATCC HTB-38) and MUC2 proteins (M2378, Sigma) was performed as described previously (Couvigny et al., 2017). Experiments were repeated at least three times, each in duplicate.

### Electron Microscopy

Transmission and scanning electron microscopy (TEM and SEM, respectively) experiments were conducted as described previously (Couvigny et al., 2017). Images were acquired and analyzed at the MIMA2 microscopy and imaging platform^3^.

### Auto-aggregation and Co-aggregation Assays

The ability of bacterial cells to auto-aggregate and co-aggregate was assessed by a spectrophotometric procedure (Handley et al., 1987). Stationary-phase cells from overnight cultures were centrifuged, washed twice with PBS and resuspended in aggregation buffer to an optical density at 600 nm (OD_600_) of 0.5 ± 0.05 (Burton et al., 2013). To determine percentage auto-aggregation, bacterial suspensions were placed in a glass test tube, vortexed, and the suspensions immediately transferred to a cuvette at room temperature (20 ± 1°C). The OD_600_ of the bacterial suspensions was monitored at various time points and the percentage auto-aggregation was expressed as follows: (1 - OD_t_/OD_t0_) × 100, where OD_t_ represents the OD_600_ of the suspension at time *t* (4, 20, or 24 h) and OD_t0_ the OD_600_ at time zero. To evaluate the impact on auto-aggregation of various pre-treatments of bacteria, cell suspensions prepared in aggregation buffer as described above were treated with EDTA (2 mM, instantaneous), Ca^2+^ (CaCl2, 2 mM, instantaneous), proteinase K (0.1 M, 2 h, 37°C), trypsin (0.1 M, 2 h, 37°C) and by heat (85°C, 10 min). Proteinase K- and trypsin-treated cells were harvested by centrifugation and the pellets washed twice in aggregation buffer before the auto-aggregation assay. To determine percentage co-aggregation, suspensions of *S. salivarius* strains were combined with an equal volume of a test strain (*F. nucleatum*) or aggregation buffer (as a control) and incubated at room temperature, without agitation, for at least 6 h. The percentage co-aggregation was calculated using the following equation: 100 × [OD_600_ (*S. salivarius* control + *F. nucleatum* control)/2 - OD_600_ (*S. salivarius* + *F. nucleatum*)]/OD_600_ (*S. salivarius* control + *F. nucleatum* control)/2, where OD_600_ (*S. salivarius* control + *F. nucleatum* control) represents the OD_600_ of control tubes containing only the *S. salivarius* or *F. nucleatum* strain at time *t* and OD_600_ (*S. salivarius* + *F. nucleatum*) represents the OD_600_ of the mixture of both suspensions at time *t*. Experiments were repeated at least three times, each in duplicate.

### Biofilm Formation

Biofilms were grown in microscope grade μclear^®^ base 96-well plates (Greiner Bio-one, France) and analyzed by confocal laser scanning microscopy (CLSM) as described previously (Couvigny et al., 2015b). Briefly, surface-associated bacteria were fluorescently labeled with 5 μM Syto9 (Invitrogen, France) and analyzed under a Leica SP2 CLSM at the MIMA2 platform. Cells labeled with Syto9 were exposed to an argon laser beam, set to 20% of its maximal power, at 488 nm. The resulting fluorescence was collected in the 500–550 nm range with a photomultiplier. Images (512 pixels × 515 pixels) were recorded through a water immersion long distance objective (NA = 0.8) at micrometer intervals through the thickness of the biofilm. Three independent experiments were performed for each strain. Three-dimensional projections of the biofilm structures were reconstructed using the Easy 3D function of the IMARIS 7.0 software (Bitplane, Switzerland). Extraction of quantitative biofilm geometric descriptors from the CSLM series was performed with the PHLIP Matlab-based image analysis toolbox (Mueller et al., 2006).

### Statistical Analysis

Variables are expressed as the means and standard deviations of results obtained from three (auto-aggregation, co-aggregation, and adhesion assays) or two (CLSM analysis) independent experiments. Comparisons between groups of variables were analyzed using the student’s *t*-test. GraphPad Prism version 6 (GraphPad software Inc., La Jolla, CA, United States) was used for statistical analyses. A *P*-value < 0.05 was considered to be statistically significant.

## Results

### Auto-aggregation of *S. salivarius*

**FIGURE 1:**
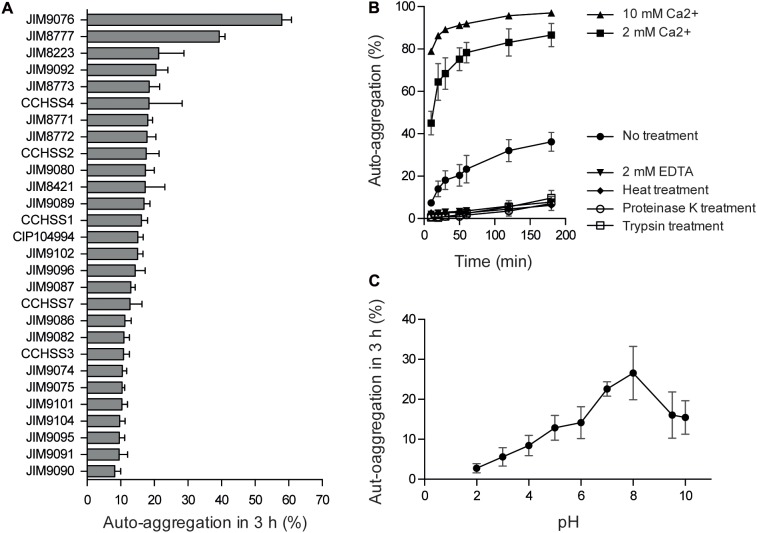
Diversity and environmental variation of the auto-aggregation phenotype in *S. salivarius*. **(A)** Auto-aggregation abilities of *S. salivarius* isolates. **(B,C)** Impact of various environmental factors on the auto-aggregation ability of *S. salivarius* JIM8777.

Twenty-eight representative strains of *S. salivarius* were screened for auto-aggregation (**Figure 1A** and **Table 1**). Most of the *S. salivarius* isolates tested under our culture conditions formed aggregates, although their auto-aggregation ability was found to be highly strain specific (ranging from 9 to 56%). We chose one of the most strongly aggregating strains, JIM8777, for further study. The chromosome sequence of JIM8777 has already been determined (Guedon et al., 2011).

To characterize the environmental factors influencing JIM8777 auto-aggregation and to clarify the nature of the interactions, we evaluated the impact of various treatments on auto-aggregation levels. Auto-aggregation was most efficient at pH 8 (**Figure 1B**). We also found that addition of Ca^2+^ led to an increase in auto-aggregation, whereas heat-treatment, addition of EDTA, proteinase K or trypsin led to a reduction in auto-aggregation (**Figure 1C**), suggesting that proteins are involved in these cell-to-cell interactions.

### Use of Semi-liquid Medium to Isolate *S. salivarius* JIM8777 Mutants with Altered Auto-aggregation Phenotypes

To identify JIM8777 genes involved in auto-aggregation, we first isolated spontaneous mutants, which exhibited increased or decreased auto-aggregation phenotype. For this, we used a strategy initially developed for selection of mutants with alterations in their ability to form chains. This method is based on differential sedimentation of bacteria in semi-liquid (SL) agar medium according to chain-forming capacity (Mercier et al., 2000, 2002; Chapot-Chartier et al., 2010). In these conditions viscosity of SL medium restricts free displacements of bacteria and sedimentation represents the main means of displacement for bacteria which do not have properties of autonomous motility. When colony of non-chain-forming bacteria initiates from one cell, simultaneous growth and sedimentation results in streaked colony (Llull et al., 2005). Contrariwise, chain-forming bacteria in these conditions are trapped in extracellular agar matrix and do not sediment, consequently forming round colonies. It is possible to observe and to select mutants escaping such immobilization as they form faster sedimenting “roots” from round colonies (Mercier et al., 2002).

**FIGURE 2:**
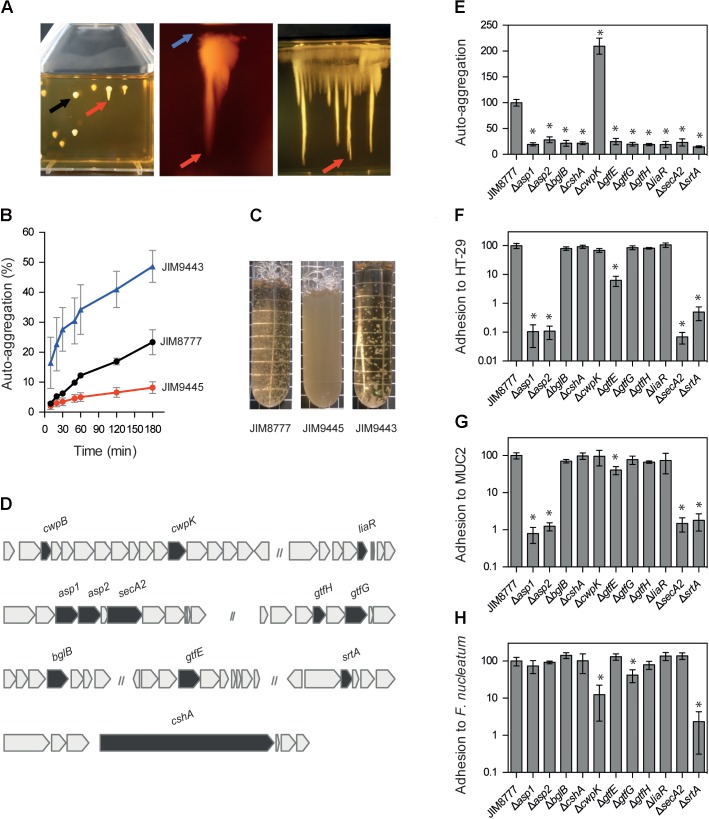
Identification of factors involved in *S. salivarius* JIM8777 auto-aggregation. **(A)** Growth of *S. salivarius* JIM8777 in semi-liquid (SL) agar medium (Left) and selection of spontaneous (Middle) and IS-induced (Right) mutants. Black arrow indicates round colonies formed by the auto-aggregating wild-type cells in SL conditions. Red and blue arrows indicate faster-sedimenting and slower-sedimenting mutants appearing as hanging down roots and as caps from round colonies, respectively. **(B,C)** Auto-aggregation abilities of the wild-type (JIM8777) and two spontaneous sedimenting mutants in liquid medium. Faster- (red, JIM9445) and slower-sedimenting (blue, JIM9443) mutants in SL agar medium correspond to non- and hyper-auto-aggregation mutants in liquid medium, respectively. **(D)** Organization of the genes involved in auto-aggregation. Mutated genes are in gray. The lengths of the genes and intergenic regions are drawn to scale. **(E)** Auto-aggregation and adhesion of the wild-type JIM8777 and reconstructed auto-aggregation mutants to the **(F)** HT-29 epithelial cell line, **(G)** MUC2 glycoprotein and **(H)** *Fusobacterium nucleatum*. Means and standard deviation values correspond to at least three independent experiments done in duplicate. Results were normalized against those of JIM8777. Asterisks indicate statistical significance (Student’s unpaired *t*-test: ^∗^*P* < 0.005).

We hypothesized that similarly to chain-forming bacteria, the auto-aggregates of JIM8777 strain will counteract sedimentation, resulting in formation of round colony. In keeping with this reasoning, we found that the auto-aggregates of the JIM8777 strain became immobilized in the SL medium (**Figure 2A**, left panel, black arrow). After 140 h of growth, we observed the appearance of “roots” and “caps” from these round colonies, presumably formed by faster- and slower-sedimenting spontaneous mutants, respectively (**Figure 2A**, left and middle panels, red and blue arrows). Samples of the roots and of the caps were then removed and streaked out on agar plates to isolate single colonies of SL medium sedimenting mutants. Analysis of bacterial growth in liquid medium confirmed that selected mutants exhibited altered auto-aggregation phenotypes (representative images of the two groups of mutants are presented in **Figures 2B,C**). Compared to the parental strain JIM8777, the slower-sedimenting mutants (such as JIM9443) showed a hyper-auto-aggregating phenotype in liquid medium, whereas the faster-sedimenting mutants (including JIM9445) showed diminished auto-aggregation capacity (**Figures 2B,C**).

We then performed a similar experiment in which bacterial cells containing the whole genome-wide random insertion library of *S. salivarius* (obtained after pGhost9::IS*S1* mutagenesis) was inoculated on the top of a flask with SL medium. After 140 h, the faster-sedimenting “roots” appeared and were collected for further analysis (**Figure 2A**, right panel). This experiment led to the isolation of 40 additional faster-sedimenting mutants.

### Identification of Auto-aggregation Factors of *S. salivarius* JIM8777

The gene mutations potentially associated with the altered sedimentation and auto-aggregation phenotypes were mapped by whole genome sequencing of 6 spontaneous mutants and cloning and sequencing of the chromosomal DNA fragments flanking the insertion site of IS*S1* from 11 insertion mutants. Altogether, 12 genes were identified from the two independent experiments (**Figure 2D** and **Table 2**). Two different spontaneous mutations were identified in the *asp1* gene, as well as two different IS*S1* insertions in the genes *cshA*, *secA2*, *gtfG* and *gtfH*. Our independent selection of several mutations in the same gene confirms the importance of these genes in the auto-aggregation phenotype.

**Table 2:** Auto-aggregation-associated genes of *S. salivarius* JIM8777.

Locus tag	Gene	Encoded protein function	Cellular function
**Spontaneous mutants**			
SalivA_1034	*cwpB*	Tyrosine-protein phosphatase	Cell wall polysaccharides synthesis
SalivA_1043	*cwpK*	Polysaccharide polymerase	Cell wall polysaccharides synthesis
SalivA_1467^(a)^	*asp1*	Putative accessory secretory protein Asp1	Protein transport
SalivA_1466	*asp2*	Putative accessory secretory protein Asp2	Protein transport
SalivA_1496	*liaR*	DNA-binding response regulator	Transcription regulation
**Insertional mutants**			
SalivA_0390	*gtfE*	Glycosyltransferase, family 4	Biosynthetic process
SalivA_0826	*bglB*	Secreted glucan binding protein	Unknown
SalivA_0893^(a)^	*cshA*	LPXTG-containing surface protein	Unknown
SalivA_1273	*srtA*	Sortase A	Modification of surface proteins
SalivA_1464^(a)^	*secA2*	Protein translocase subunit SecA2	Protein transport
SalivA_1698^(a)^	*gtfG*	Glycosyltransferase, family 4	Biosynthetic process
SalivA_1700^(a)^	*gtfH*	Glycosyltransferase, family 2	Biosynthetic process


*^(*a*)^Two mutants with different point mutations were obtained in this gene.*

To confirm the phenotypes of the bacterial mutants and the correlation between sedimentation in SL conditions and auto-aggregation in liquid medium, we constructed a series of mutants in the JIM8777 background carrying deletions of the genes in which we identified the point mutations associated with altered sedimentation (**Table 1**). We found that deletion of the *asp1*, *asp2*, *bglB*, *cshA*, *gtfE*, *gtfG*, *gtfH*, *liaR*, *secA2*, and *srtA genes*, point mutations in which induced a faster-sedimenting phenotype in SL conditions, led to a decreased auto-aggregation phenotype in liquid medium (**Figure 2E**). As *cwpB* and *cwpK*, two genes coding for enzymes predicted to synthetize cell wall polysaccharides (WPS), are located in the same operon (**Figure 2D**), we chose to include only one of these genes (*cwpK*) for further analyses and confirmed that the Δ*cwpK* mutant showed an increased auto-aggregation phenotype (**Figure 2E**). Altogether, our results confirmed that deletion of the genes identified as having an impact on bacterial sedimentation also affected the auto-aggregation properties of *S. salivarius*. This confirms that our method, based on differential sedimentation in SL conditions, provided an efficient tool for the specific isolation of mutants with altered auto-aggregation properties.

### Capacity of JIM8777 Derivatives to Adhere to Host Surfaces and to Co-aggregate with *Fusobacterium nucleatum*

We investigated the ability of the auto-aggregation mutants to adhere to the human colonic epithelial cell line HT-29 (**Figure 2F**), to the mucin 2 (MUC2) glycoprotein (**Figure 2G**) and to the human pathogen *F. nucleatum* (co-aggregation) (**Figure 2H**). In comparison to the wild-type JIM8777, 5 out of 10 of the deletion mutants with altered auto-aggregation capacity (Δ*asp1*, Δ*asp2*, Δ*gtfE*, Δ*secA2*, and Δ*srtA*) displayed a significant (*P* < 0.005) reduction in adhesion to both HT-29 and MUC2 (**Figures 2F,G**). Moreover, the *cwpK* (increased auto-aggregation) and *srtA* (decreased auto-aggregation) deletion strains showed a drastic reduction (>90%; *P* < 0.0001) in co-aggregation with *F. nucleatum* compared to the wild-type strain (**Figure 2H**). The co-aggregation ability of the Δ*gtfG* strain was also altered but to a lesser extent (50% reduction). Similar results were obtained with the spontaneous mutants *asp1*_*spont*1_ (JIM9445), *asp2_spont_* (JIM9447), *cwpB_spont_* (JIM9442) and *cwpK_spont_* (JIM9443) (data not shown).

### Role of JIM8777 Auto-aggregation Factors in Biofilm Development

**FIGURE 3:**
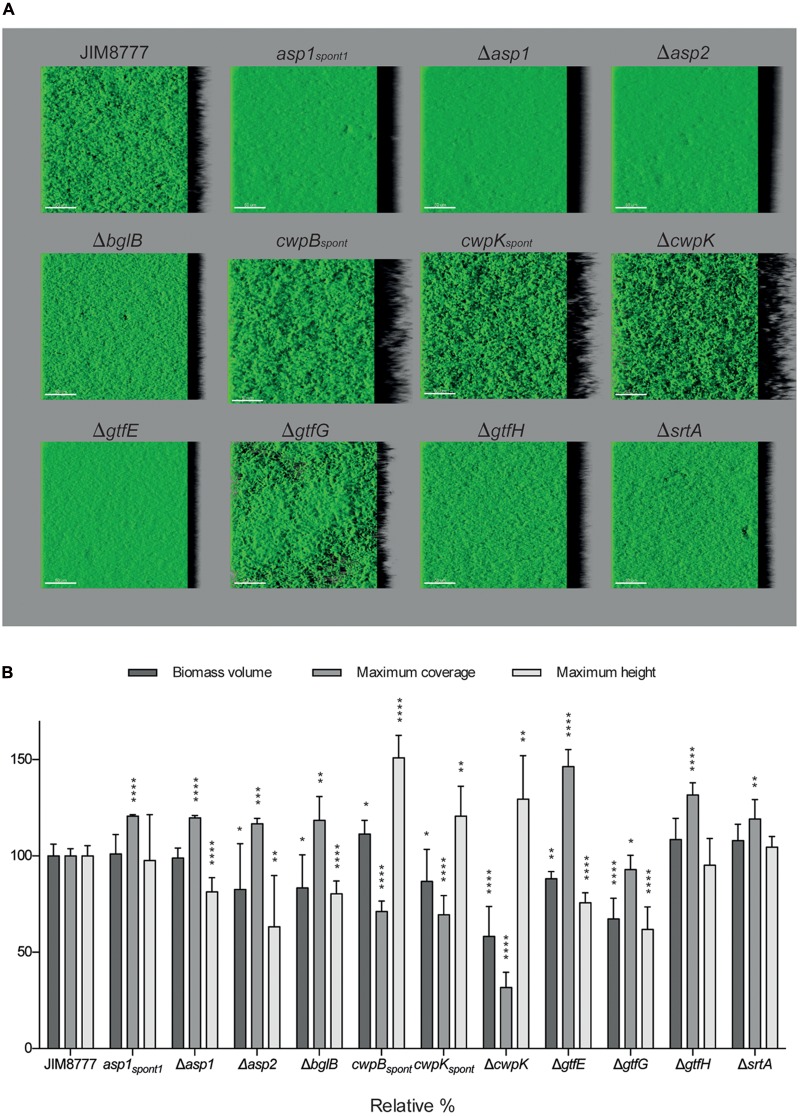
Auto-aggregation factors of JIM8777 and their impact on biofilm architecture. **(A)** 3-D reconstructions of representative confocal images of 5-h-old biofilms formed by *S. salivarius* strains. Scale bars: 50 μm. **(B)** Biomass volume (μm^3^), maximum coverage (%) and maximum height (μm) were normalized against values obtained for JIM8777 and measured for each strain in nine different CLSM image stacks from two independent experiments. Student’s *t*-test was used for statistical analysis (^∗∗∗∗^*P* < 0.0001, ^∗∗∗^*P* < 0.0005, ^∗∗^*P* < 0.005, ^∗^*P* < 0.05).

Cell aggregation and biofilm formation are interconnected bacterial properties (Sanchez et al., 2010; Couvigny et al., 2017). We evaluated whether the auto-aggregation factors we had identified also contributed to biofilm formation by analyzing biofilms formed by JIM8777 derivatives and comparing them with those of the wild-type strain by confocal microscopy. After 5 h of sessile growth, all mutants were able to form biofilms under the conditions employed. JIM8777 formed a relatively dense and filamentous biofilm that covered the entire available surface (**Figure 3**). Biofilms formed by the mutants differed in their architecture from JIM8777 (*P* < 0.05 compared to wild-type for at least one geometric descriptor of the biofilm structure). With the exception of strain Δ*gtfG*, all non-auto-aggregative strains (*asp1*_*spont*1_,Δ*asp1*,Δ*asp2*, Δ*bglB*,Δ*gtfE*,Δ*gtfH*,Δ*srtA*) developed compact and uniform biofilms. Hyper-auto-aggregative mutants (*cwpB_spont_, cwpK_spont_* and Δ*cwpK*) formed excessively filamentous biofilms compared to wild-type. This result indicates that the *S. salivarius* factors implicated in cell-to-cell interactions in planktonic conditions also influence the morphology and spatial organization of bacterial biofilm communities.

### Evaluation of the Cell Surface Morphology of the *cwpK* and *asp1* Mutants by Electron Microscopy

**FIGURE 4:**
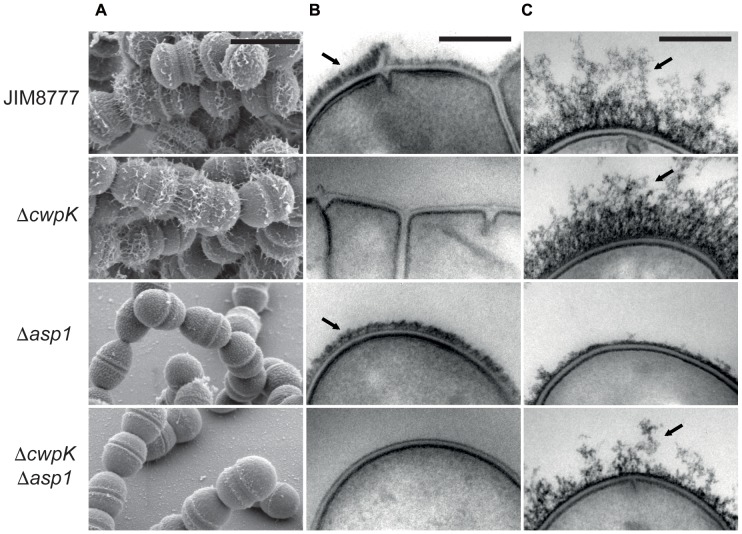
Surface structure of *S. salivarius* strains. **(A)** Scanning electron micrographs of *S. salivarius* strains. Scale bars: 1 μm. **(B)** Transmission electron micrographs of osmium tetroxide-treated *S. salivarius* strains. Scale bars: 0.2 μm. **(C)** Transmission electron micrographs of osmium tetroxide- and ruthenium red-treated *S. salivarius* strains. Black arrows indicate cell wall polysaccharide **(B)** and the glycoproteinaceous fibril-like structures **(C)**. Scale bars: 0.2 μm.

To provide insight into the role played by the products of the *cwpK* and *asp1* genes in auto-aggregation, we evaluated the cell surface morphology of the corresponding mutants by scanning and transmission electron microscopy (SEM and TEM, respectively). We observed by SEM that the JIM8777 cells exhibited a rough and irregular surface that was partially covered by fibril-like structures (**Figure 4A** and Supplementary Figure S1). The Δ*cwpK* mutant displayed a smooth surface with fibril-like structures, whereas the Δ*asp1* mutant exhibited a rough surface without fibrils. Furthermore, the Δ*cwpK* Δa*sp1* mutant exhibited an additive phenotype, with both a smooth and bald surface. These findings suggest that surface roughness is linked to WPS biosynthesis involving CwpK, whereas Asp1 may be responsible for the presence of the extracellular fibrils.

TEM of osmium tetroxide-stained cells of the wild-type strain revealed the presence of an electron dense layer outside the cell wall, potentially corresponding to WPS (**Figure 4B**). The overall morphology of JIM8777 and Δa*sp1* was similar, however, the electron-dense layer was completely absent in micrographs of the Δ*cwpK* and Δ*cwpK* Δ*asp1* strains. A similar result was obtained with the spontaneous *cwpK_spont_* (JIM9443) mutant, confirming that the point mutation resulted in loss of function (Supplementary Figure S2).

We have shown previously that the SecA2/Y2 transported glycoproteins form fibril-like structures that can be observed outside of the cell wall of ruthenium red-stained cells (Couvigny et al., 2017). Accordingly, here we found that the fibrillar layer (mainly composed by SRR glycoproteins) was greatly diminished in the Δ*asp1* and Δ*cwpK*Δ*asp1* mutants (**Figure 4C**), as well as in the *asp1*_*spont*1_ and Δ*srtA* strains (Supplementary Figure S2). Altogether, these results indicate that CwpB and CwpK are involved in WPS production and show that Asp1 and SrtA play a role in fibril detection, and therefore in the SRR glycoprotein synthesis pathway. It should also be noted that the surface glycoprotein layer appeared denser in the *cwpK* mutant background (i.e., Δ*cwpK* and Δ*cwpK*Δ*asp1*), suggesting that WPS may hamper glycoprotein formation, exposure, or detection.

## Discussion

In this study, we show that auto-aggregation is a property of many isolates of the pioneer colonizer and commensal bacterium *S. salivarius*. Auto-aggregation is influenced by a variety of environmental factors and involves bacterial proteins (**Figure 1**). To identify the proteins determining cell-to-cell interactions of a highly auto-aggregating *S. salivarius* strain (JIM8777), we developed a screening strategy based on immobilization of cell clusters in SL medium that allowed us to select for mutants with altered auto-aggregation. Twelve proteins involved in different aspects of auto-aggregation in *S. salivarius* were identified. It has been shown previously that the surface-exposed adhesin, SrpB, mediates auto-aggregation in *S. salivarius* JIM8777 (Couvigny et al., 2017). Our screen led us to identify mutations in several genes coding for components of the maturation and secretion pathway of SrpB that had a negative impact on the bacterial auto-aggregation phenotype: the SecA2 motor protein, the Asp1 and Asp2 chaperones, the GtfE glycosyltransferase and the SrtA sortase. We also demonstrated that the SrtA sortase is directly involved in SRR glycoprotein surface exposure. Redundancy of mutations in the SrpB pathway confirmed the importance of this pathway in *S. salivarius* auto-aggregation.

New determinants of *S. salivarius* auto-aggregation were also identified by our screening method in SL medium: CshA, BglB, CwpB, CwpK, GtfG, GtfH and LiaR. Homologs of these genes in other streptococcal species have already been reported to contribute to auto-aggregation. The *Streptococcus gordonii* adhesin CshA exhibits auto-aggregative properties (McNab et al., 1999) and the BglB homolog in *Streptococcus mutans* contributes to auto-aggregation by linking bacterial cells and extracellular molecules of glucan (Shah and Russell, 2004; Lynch et al., 2007).

Two enzymes (CwpB and CwpK), which are required for WPS biosynthesis in *S. salivarius* JIM8777, also participate in auto-aggregation. However, in contrast to the other genes identified, mutations in *cwpB* and *cwpK* caused an increase in auto-aggregation. A similar increase in auto-aggregation has already been associated with mutations in the WPS locus of *Streptococcus mitis* (Rukke et al., 2012). It is possible that WPS in JIM8777 blocks surface accessibility to *S. salivarius* adhesins, as demonstrated for WPS in *Streptococcus pneumoniae* (Sanchez et al., 2011). Interestingly, we found that the surface of *S. salivarius cwp* mutants displayed a much denser external fibrillar layer than the wild-type strain JIM8777, also suggesting that WPS could interfere with surface exposure of auto-aggregation promoting cell surface factors, such as SrpB.

We also identified two additional *S. salivarius* auto-aggregation factors: the GtfG and GtfH glycosyltransferases. Glycosyltransferases participate in the maturation and/or synthesis of various extracellular compounds, including glucans, cell wall polysaccharides, lipoteichoic acid, peptidoglycan and glycoproteins. Therefore, mutations in genes encoding the GtfG and GtfH glycosyltransferases may affect some of these compounds, leading to consequences for the auto-aggregation capacity of these mutants.

The ability of bacterial cells to form auto-aggregates is considered as an advantageous feature under certain environmental conditions, however, excessively large cell clusters may increase the probability of their clearance (Koop et al., 1989). This suggests that bacteria have finely tuned regulatory mechanisms to ensure optimal levels of auto-aggregation. In this study, we provide evidence that auto-aggregate formation is both positively and negatively modulated by various environmental factors and several bacterial components, indicating that their formation is a dynamic and controlled process. LiaR may be part of this regulatory network: we found mutations in the gene encoding this protein led to decreased auto-aggregation. LiaR homologs are implicated in the cell wall stress response and control expression of several surface proteins (Suntharalingam et al., 2009; Klinzing et al., 2013), which may have an effect on auto-aggregation. Our results indicate that auto-aggregation is a multifactorial process in *S. salivarius* but further investigations are required for a more comprehensive assessment of how these factors act and interplay.

Auto-aggregation measurements are widely used as an indirect method for evaluating the adhesion capacity of bacteria, including potential probiotics (Saeed and Heczko, 2007; Walter et al., 2008; Xu et al., 2009; Piwat et al., 2015). In line with this strategy, we identified mutations in genes that also affect adhesion of *S. salivarius* to host surfaces, such as MUC2 and HT-29 cells (i.e., *asp1*, *asp2*, *gtfE*, *secA2*, and *srtA*), and co-aggregation with the human pathogen *F. nucleatum* (i.e., *cwpK*, *gtfG*, *srtA*). As mentioned above, these mutations impact the SRR glycoprotein synthesis pathway. SRR glycoproteins, which are important for colonization and persistence in mice, also mediate adhesion of *S. salivarius* to host cells and host-derived molecules, such as MUC2 (Couvigny et al., 2017). In addition to their effect on SRR glycoproteins, GtfE glycosylates numerous other extracellular proteins in *S. salivarius* (Couvigny et al., 2017) and the genome of *S. salivarius* JIM8777 encodes 23 potential SrtA substrates (i.e., LPTXG-containing proteins) (Delorme et al., 2015). The activity of SecA2, Asp1 and Asp2 is strictly dedicated to SRR glycoprotein synthesis, whereas SrtA and GtfE may have a much broader role and additional proteins, other than those transported by the SecA2/Y2 system, could be involved in the adhesive phenotypes observed in this study.

We also identified mutations affecting co-aggregation with *F. nucleatum*, a multifaceted bacterium that plays a pivotal role in oral biofilm community development (Kolenbrander and London, 1993) and is associated with periodontal disease and colon cancer (Han, 2015). It has been shown that co-aggregation results predominantly from the specific interaction of a cell-surface protein of a given/specific bacterium with a polysaccharide receptor expressed on the surface of a second bacterium (Rickard et al., 2003). The requirement for this type of interaction would explain the decreased co-aggregation phenotype of *cwpB* and *cwpK* mutants of *S. salivarius*. It also suggests that the WPS of *S. salivarius* act as receptors for the binding of *F. nucleatum*. This explanation is supported by published data showing that WPS expressed by other oral bacteria are implicated in co-aggregation with *F. nucleatum* (Rosen and Sela, 2006). In addition to mutations in WPS genes, co-aggregation was also decreased in the *srtA* and *gtfG* mutants, indicating the existence of at least one more *S. salivarius* determinant that remains to be identified. This determinant is likely to be a glycosylated SrtA-anchored surface protein, which may function as an adhesin. This unknown protein is not transported by the SecA2Y2 system and is not a LPXTG-containing CshA or SRR protein. Thus, *S. salivarius* JIM8777 appears to express both a cell surface adhesin and a carbohydrate receptor specific for the interaction with *F. nucleatum*. This may provide a partial explanation for the strain specificity of the co-aggregation reaction (Weerkamp and McBride, 1980; Handley et al., 1987; Arzmi et al., 2015; Barbosa et al., 2015; Lima et al., 2017).

In summary, we have identified several auto-aggregation factors from our screen of a random insertion mutant library and selection of spontaneous mutants in SL medium. All of these factors influence biofilm architecture, demonstrating that cell–cell interactions governing auto-aggregation and biofilm development are interconnected. Some of these factors also contribute to the adhesion of *S. salivarius* to host biological substrates, including MUC2, the human colonic epithelial cell line HT-29, and *F. nucleatum*. These auto-aggregation factors may exert key functions in terms of human health and disease by playing roles in bacterial colonization and persistence, microbial community development and pathogen elimination. Identification of these factors paves the way for further molecular studies. In addition, our results demonstrate that the screening method developed in this study can be used to identify the genes involved in bacteria-bacteria and bacteria–host interactions. This method could be especially useful if applied to isolates, for which molecular biology tools have not yet been developed. Our screen may also facilitate molecular studies of microbial community development of pathogenic or probiotic bacteria, for which aggregation is either a virulence trait or beneficial characteristic, respectively.

## Author Contributions

SK and EG designed the experiments and wrote the manuscript. BC, SK, NP, BQ, A-LA, CD, TM, RB, NL, and EG performed the experiments. BC, SK, NP, A-LA, RB, PR, NL, and EG performed the analyses. All of the authors critically reviewed the manuscript and approved the final version.

## Conflict of Interest Statement

The authors declare that the research was conducted in the absence of any commercial or financial relationships that could be construed as a potential conflict of interest.

## Footnotes

1 http://www.i2bc.paris-saclay.fr/

2 http://mgps.eu/

3 http://www6.jouy.inra.fr/mima2
