# Supplementary material for: Identification of New Factors Modulating Adhesion Abilities of the Pioneer Commensal Bacterium Streptococcus salivarius
Source: Front Microbiol. 2018 Feb 20;9:273. doi: 10.3389/fmicb.2018.00273 (PMC5826255; doi:10.3389/fmicb.2018.00273)
Supplement: Supplementary file 1 [file Table_1.PDF]

Table S1. Primers used in this study.

Primer	Sequence (5’-3’)	DNA target[a]	Name of constructed strain	Source
Primers used to amplify EryR or KanR cassettes.				
EG940	5’-TGTAATTAAGAAGGAGTGA-3’	pGhost9		(1)
EG941	5’-TAGAATTATTTCCTCCCGT-3’			
EG1265	5’-AGCTTCTTGGGGTATCTTTAAATACTGT-3’	pKa		(2)
EG1266	5’-TTAGACATCTAAATCTAGGTACTA-3’			
Primers used to create overlap PCR products containing the EryR or KanR cassette. The PCR products are then transformed to create mutant strains.				
EG1479	5’-TTGTACCAGCATGCAGAGACGAGA-3’	Upstream SALIVA_0826		
EG1480	5’-TCATGTAATCACTCCTTCTTAATTACACACATATACTTTAACTTTTTGGAACATAGT-3’		JIM9478	This study
EG1481	5’-TATTTAACGGGAGGAAATAATTCTAATGAGTGCACAAAGGCACTGAAGAAGATGA-3’	Downstream SALIVA_0826		
EG1482	5’-TCTTTAGTAATGTAGCCCATGCGA-3’			
EG1485	5’-TGAATCAGGTAACACTGCTCGTTTGAT-3’	Upstream SALIVA_0893		
EG1486	5’-TCATGTAATCACTCCTTCTTAATTACAAAATAAATCTTTACCCACTTTGGTAATCCT-3’		JIM9482	This study
EG1487	5’-TATTTAACGGGAGGAAATAATTCTAGGTACAAAACGACGCAAACGTTAAAATGAT-3’	Downstream SALIVA_0893		
EG1488	5’-TAGAGGTACCAGTAACATTGCCTT-3’			
EG1497	5’-TCAAGACCGTCGTGCTAGTGTCATTT-3’	Upstream SALIVA_1698		
EG1498	5’-TCATGTAATCACTCCTTCTTAATTACAGTCCACACCATTAGGCTCAAAGCCCACCAA-3’		JIM9490	This study
EG1499	5’-TATTTAACGGGAGGAAATAATTCTACTCTTGATAGCCATACGATAAGTGTTTTGA-3’	Downstream SALIVA_1698		
EG1500	5’-ATAAGTAGGTTTGAGACCAACAAT-3’			
EG1510	5’-TTTCCGTTATGCGATTCCTGAGTTTGTT-3’	Upstream SALIVA_1700		
EG1511	5’-TCATGTAATCACTCCTTCTTAATTACAATTTTCCATGGTCTATCTCTCAATCGT-3’		JIM9492	This study
EG1512	5’-TATTTAACGGGAGGAAATAATTCTATTGGAGCAATAAAGGTCGAGGGTTAAGCTA-3’	Downstream SALIVA_1700		
EG1513	5’-ATGTTGCATCAGATACTTCACTTCCAT-3’			
EG1528	5’-ACAGTCGTGAGCTCAATCTTACTTTAGGA-3’	Upstream SALIVA_0390		
EG1529	5’-TCATGTAATCACTCCTTCTTAATTACATCCTAAATTGATGTTATAAACAGTCATGTA-3’		JIM9486	This study
EG1530	5’-TATTTAACGGGAGGAAATAATTCTAAACTTAGTAAAGGAGATGACTAGTCATGATTAAT-3’	Downstream SALIVA_0390		
EG1531	5’-AAGACGCTTAACCTGCTCTGTTGTAATATTA-3’			
EG1552	5’-ATGCTCGTCGTACAGAACTTATGGTCGGA-3’	Upstream SALIVA_1273		
EG1553	5’-TCATGTAATCACTCCTTCTTAATTACATTTGCTCATTAGTTGTCACCACTTTCTTGG-3’		JIM9496	This study
EG1555	5’-TATTTAACGGGAGGAAATAATTCTAAATCAATTTCAAAGTTAAGAAAGGGGCCTT-3’	Downstream SALIVA_1273		
EG1556	5’-TTGCGGTGTCACAATCTCTACACTCAA-3’			
EG1307	5’-GAGCCTCATGCCATCATCATGTCT-3’	Upstream SALIVA_1464		
EG1308	5’-TCATGTAATCACTCCTTCTTAATTACACAGTTGTTTAAACATGTTCTTCTCCTG-3’		JIM9303	This study
EG1248	5’-TATTTAACGGGAGGAAATAATTCTAAAAGGAGATATCATCAGCCATTTCCCATAA-3’	Downstream SALIVA_1464		
EG1249	5’-TATGAACCTCAATCACCGCACGA-3’			
EG1242	5’-TGTTCAATCTTTGGCAGTATCCTT-3’	Upstream SALIVA_1467		
EG1243	5’-TCATGTAATCACTCCTTCTTAATTACAAAAATAATACATGAGTTCTCCTTCATT-3’		JIM9310	This study
EG1293	5’-TATTTAACGGGAGGAAATAATTCTATGGTTGAAGGAGGAGCAGAATGACCAAGGA-3’	Downstream SALIVA_1467		

EG1397	5’-TCAAGCTCTTCAGTTCCCATATAGAAG-3’			
EG1174	5’-TTGTAGTGATGGATAGCTTTAACCT-3’	Upstream SALIVA_1496		
EG1175	5’-TCATGTAATCACTCCTTCTTAATTACACTTATTCGACATTGTCTTCTCCT-3’		JIM9471	This study
EG1176	5’-TATTTAACGGGAGGAAATAATTCTAGATGATGAATAAAGCTATCAACTA-3’	Downstream SALIVA_1496		
EG1177	5’-ACTTAACGATATCAAAGTTCT-3’			
EG1631	5’-TTATCTACTTCGTTGATAGTGACGAT-3’	Upstream SALIVA_1043		
EG1632	5’-TCATGTAATCACTCCTTCTTAATTACAACTGCTATTCACAAGTACTTTCCATACCTT-3’		JIM9465 and JIM9469	This study
EG1633	5’-TATTTAACGGGAGGAAATAATTCTATTGTAATTGAAGCAGAGGTATATCATTTAGA-3’	Downstream SALIVA_1043		
EG1634	5’-ATCCAAAGTCACGTACAGTGAAATGGCA-3’			
EG1296	5’-TTGTAGCCAATCCTCGTACGGAAGGTCGT-3’	Upstream SALIVA_1466		
EG1297	5’-TCATGTAATCACTCCTTCTTAATTACATTTCAGGCGATTATCCTTGGTCATTCTGCT-3’		JIM9307	This study
EG1298	5’-TATTTAACGGGAGGAAATAATTCTAGATTTTGGAAGGAAGGAGTAAGAATGTTCA-3’	Downstream SALIVA_1466		
EG1299	5’-ATTGGTCGTGTAAACGATGTCTGA-3’			
EG1242	5’-TGTTCAATCTTTGGCAGTATCCTT-3’	Upstream SALIVA_1467		
EG1365	5’-ACAGTATTTAAAGATACCCCAAGAAGCTAAAATAATACATGAGTTCTCCTTCATT-3’		JIM9324 and JIM9469	This study
EG1366	5’-TAGTACCTAGATTTAGATGTCTAAGAAACAGGAGAAGAACATGTTTAAACAACT-3’	Downstream SALIVA_1465		
EG1245	5’-TTCTTGCCATCACGAATGATGTAGT-3’			
Primers used for the validation of mutant strains obtained by natural transformation.				
EG1477	5’-TCTTCATAAATACTACATGCA-3’	Upstream SALIVA_0355	JIM9477	This study
EG1478	5’-TGGTGTTAATCTAGAAGAATAT-3’	Downstream SALIVA_0355		
EG1483	5’-TTACAGTATTTGATCATGGTCT-3’	Upstream SALIVA_0826	JIM9478	This study
EG1484	5’-ATGGCTTCAATCTTACGTGCAGCA-3’	Downstream SALIVA_0826		
EG1489	5’-TCATCAACATTTGACCGTTCA-3’	Upstream SALIVA_0893	JIM9482	This study
EG1490	5’-TTGATGGTACGGCGTACTCGA-3’	Downstream SALIVA_0893		
EG1501	5’-TTGAAACGGATATTGATAAGGA-3’	Upstream SALIVA_1698	JIM9490	This study
EG1502	5’-TGCTATACGAACAGGTGCAGA-3’	Downstream SALIVA_1698		
EG1514	5’-TTACGCTGATGAATCGTCGTTTAGA-3’	Upstream SALIVA_1700	JIM9492	This study
EG1515	5’-TATAGACCGTTGGTAAGTCCTTATA-3’	Downstream SALIVA_1700		
EG1532	5’-ATGGTACAGACTACGAGCCAGTAGCA-3’	Upstream SALIVA_0390	JIM9486	This study
EG1533	5’-AGATATTACTTGGTGCGGTATATTCA-3’	Downstream SALIVA_0390		
EG1557	5’-ACTGATGCTGAGGCTCAAGCTGAGT-3’	Upstream SALIVA_1273	JIM9496	This study
EG1558	5’-ATATCAGTCATAGCTACATTA-3’	Downstream SALIVA_1273		
EG1635	5’-TGCGATACTTATGCTAGTGAA-3’	Upstream SALIVA_1043	JIM9465 and JIM9469	This study
EG1636	5’-AGACAACTCATGCATCTTGT-3’	Downstream SALIVA_1043		
EG1178	5’-ATCGGACGGTTAAATTGATT-3’	Upstream SALIVA_1496	JIM9471	This study
EG1179	5’-TCACCGTCATCAAGGTCTGTT-3’	Downstream SALIVA_1496		
EG1246	5’-TTCTCTAGTGCAATGATGGGT-3’	Upstream SALIVA_1467	JIM9324 and JIM9469	This study
EG1247	5’-TCGATGGCTTGGTGGAGTCCA-3’	Downstream SALIVA_1465		
EG1300	5’-TGATTCGCTACTATAAAGATGAGCA-3’	Upstream SALIVA_1466	JIM9307	This study
EG1301	5’-AAGGCGTCTGTGCGCTATCAAGGA-3’	Downstream SALIVA_1466		
EG1246	5’-TTCTCTAGTGCAATGATGGGT-3’	Upstream SALIVA_1467	JIM9310	This study
EG1295	5’-AGAGTCTGGCACGATTACCGA-3’	Downstream SALIVA_1467		
EG1302	5’-TTCAGCCCTGTCTTGGACTGGACCTAT-3’	Upstream SALIVA_1464	JIM9303	This study

EG1250	5’-TCGTACTGGCTATAAATGTCA-3’	Downstream SALIVA_1464	
**Primers used for IS*S1* insertion site determination.**			
HD1	5’-ACGACGGCCAGTGAGCGCGCGTA-3’	Transposon mutants	This study
HU2	5’-ACTGCCTCAACCACGATT-3’		

[a] Refer to the hybridization target of primers.

(1) Maguin E, Prevost H, Ehrlich SD, Gruss A (1996) Efficient insertional mutagenesis in lactococci and other gram-positive bacteria. J Bacteriol 178: 931-935.

(2) Débarbouillé M, Arnaud M, Fouet A, Klier A, Rapoport G (1990) The *sacT* gene regulating the *sacPA* operon in *Bacillus subtilis* shares strong homology with transcriptional antiterminators. J Bacteriol 172: 3966–3973.J Bacteriol 172: 3966–3973.
