# Supplementary material for: Identification of New Factors Modulating Adhesion Abilities of the Pioneer Commensal Bacterium Streptococcus salivarius
Source: Front Microbiol. 2018 Feb 20;9:273. doi: 10.3389/fmicb.2018.00273 (PMC5826255; doi:10.3389/fmicb.2018.00273)
Supplement: Supplementary file 2 [file Image_1.PDF]

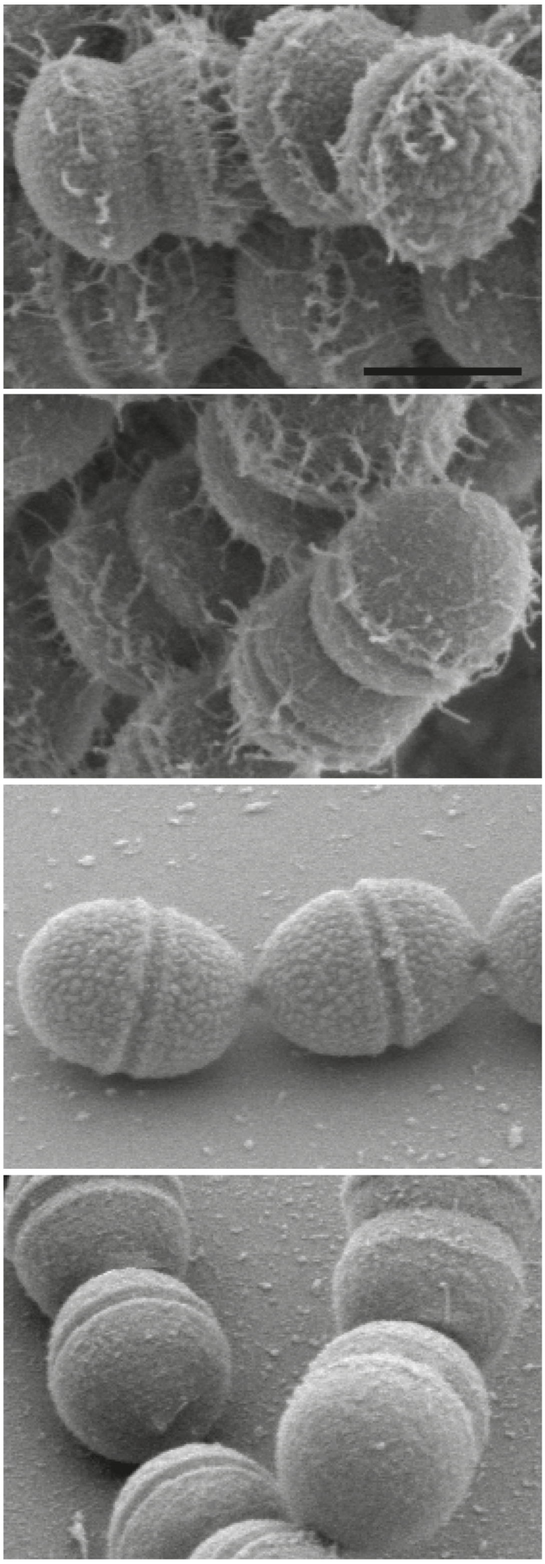

JIM8777

Δ*cwpK*

Δ*asp1*

Δ*cwpK*
Δ*asp1*

FIGURE S1. Scanning electron micrographs of *S. salivarius* strains. Scale bars: 0.5 µm.
