# Supplementary material for: Identification of New Factors Modulating Adhesion Abilities of the Pioneer Commensal Bacterium Streptococcus salivarius
Source: Front Microbiol. 2018 Feb 20;9:273. doi: 10.3389/fmicb.2018.00273 (PMC5826255; doi:10.3389/fmicb.2018.00273)
Supplement: Supplementary file 3 [file Image_2.PDF]

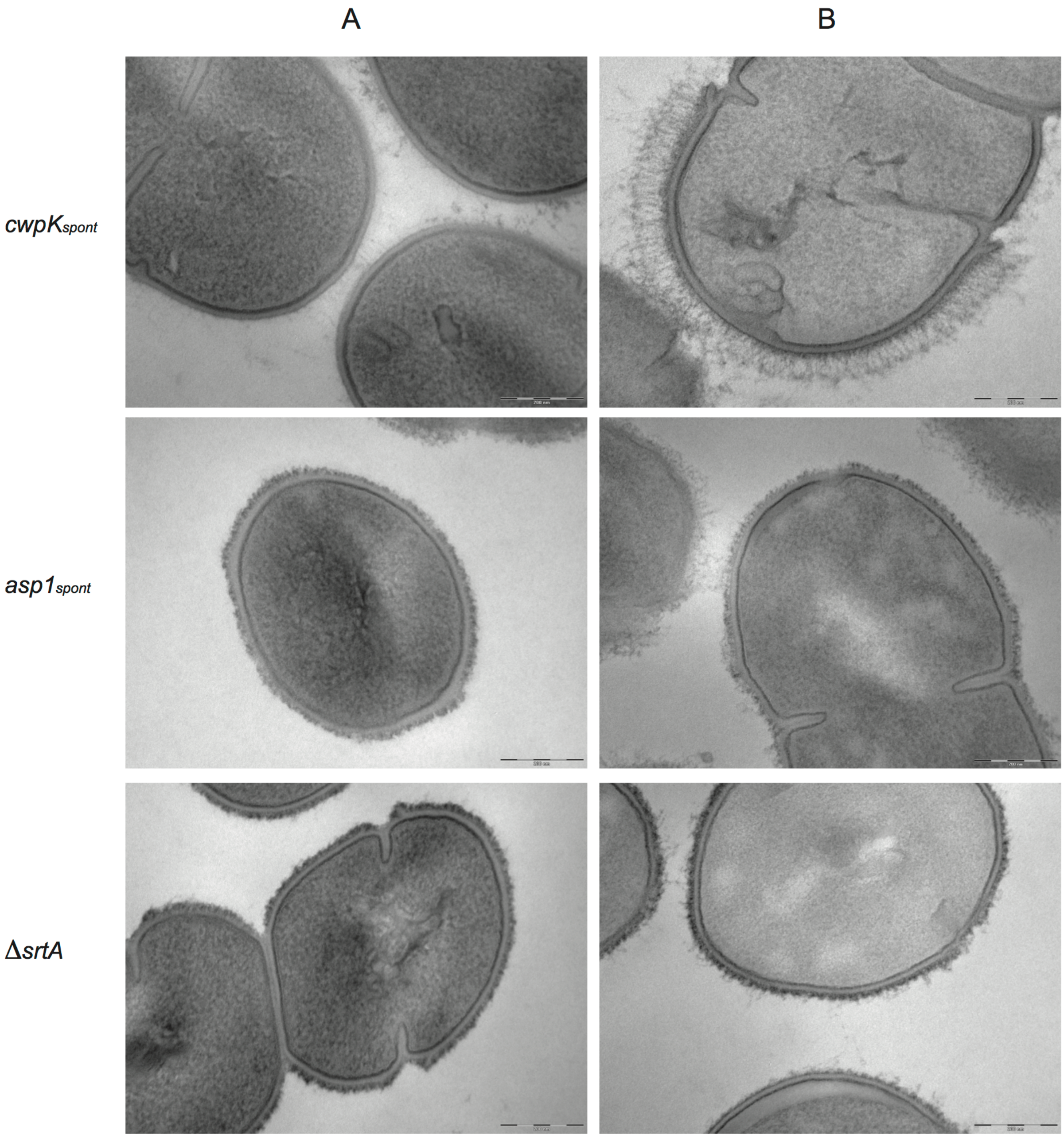

FIGURE S2. Surface structure of *S. salivarius* JIM8777 mutants. (**A**) Transmission electron micrographs of osmium tetroxide-treated JIM8777 mutants. Scale bars: 0.2 µm. (**B**) Transmission electron micrographs of osmium tetroxide- and ruthenium red-treated JIM8777 mutants. Scale bars: 0.2 µm.
